# Changes of aortic hemodynamics after aortic valve replacement—A four dimensional flow cardiovascular magnetic resonance follow up study

**DOI:** 10.3389/fcvm.2023.1071643

**Published:** 2023-02-14

**Authors:** Stephanie Wiesemann, Ralf Felix Trauzeddel, Ahmed Musa, Richard Hickstein, Thomas Mayr, Florian von Knobelsdorff-Brenkenhoff, Emilie Bollache, Michael Markl, Jeanette Schulz-Menger

**Affiliations:** ^1^Charité – Universitätsmedizin Berlin, corporate member of Freie Universität Berlin and Humboldt-Universität zu Berlin, ECRC Experimental and Clinical Research Center, Working Group Cardiovascular Magnetic Resonance, Berlin, Germany; ^2^Department of Cardiology and Nephrology, HELIOS Klinikum Berlin Buch, Berlin, Germany; ^3^DZHK (German Center for Cardiovascular Research), Partner Site Berlin, Berlin, Germany; ^4^Charité – Universitätsmedizin Berlin, corporate member of Freie Universität Berlin and Humboldt-Universität zu Berlin, Department of Anesthesiology and Intensive Care Medicine, Charité Campus Benjamin Franklin, Berlin, Germany; ^5^Clinic Agatharied, Department of Cardiology, Ludwig Maximilian University of Munich, Hausham, Germany; ^6^CNRS, INSERM, Laboratoire d’Imagerie Biomédicale (LIB), Sorbonne Université, Paris, France; ^7^Department of Radiology, Feinberg School of Medicine, Northwestern University, Chicago, IL, United States

**Keywords:** cardiovascular magnetic resonance imaging, 4D flow, aorta, aortic stenosis, bicuspid aortic valve, aortic valve replacement

## Abstract

**Objectives:**

Non-invasive assessment of aortic hemodynamics using four dimensional (4D) flow magnetic resonance imaging (MRI) provides new information on blood flow patterns and wall shear stress (WSS). Aortic valve stenosis (AS) and/or bicuspid aortic valves (BAV) are associated with altered aortic flow patterns and elevated WSS. Aim of this study was to investigate changes in aortic hemodynamics over time in patients with AS and/or BAV with or without aortic valve replacement.

**Methods:**

We rescheduled 20 patients for a second 4D flow MRI examination, whose first examination was at least 3 years prior. A total of 7 patients received an aortic valve replacement between baseline and follow up examination (=operated group = OP group). Aortic flow patterns (helicity/vorticity) were assessed using a semi-quantitative grading approach from 0 to 3, flow volumes were evaluated in 9 planes, WSS in 18 and peak velocity in 3 areas.

**Results:**

While most patients had vortical and/or helical flow formations within the aorta, there was no significant change over time. Ascending aortic forward flow volumes were significantly lower in the OP group than in the NOP group at baseline (NOP 69.3 mL ± 14.2 mL vs. OP 55.3 mL ± 1.9 mL *p* = 0.029). WSS in the outer ascending aorta was significantly higher in the OP group than in the NOP group at baseline (NOP 0.6 ± 0.2 N/m^2^ vs. OP 0.8 ± 0.2 N/m^2^, *p* = 0.008). Peak velocity decreased from baseline to follow up in the aortic arch only in the OP group (1.6 ± 0.6 m/s vs. 1.2 ± 0.3 m/s, *p* = 0.018).

**Conclusion:**

Aortic valve replacement influences aortic hemodynamics. The parameters improve after surgery.

## 1. Introduction

Four-dimensional (4D) flow cardiovascular magnetic resonance imaging (MRI) provides non-invasive blood flow visualization and quantification and can be used in cardiovascular pathologies to display and measure abnormal flow patterns and derived parameters such as wall shear stress (WSS) (1). Indeed, 4D flow MRI has shown abnormal helical and vortical flow formations and elevated WSS in the aorta of patients with a tricuspid aortic valve (TAV) and aortic stenosis (AS) or a bicuspid aortic valve (BAV) (2–4). The location and extent of these aberrances depended on the severity of the stenosis (2) or cusp fusion morphology in BAV (5). Patients with BAV already showed abnormal flow and elevated WSS even without stenosis (6) and also showed an increase in aortic peak velocities and a decrease in WSS over time (6). Additionally, abnormal flow formations and increased WSS were reported in patients who underwent aortic valve replacement (7), with distinct patterns according to the implanted valve and procedure type (8–10). Importantly, elevated WSS has been shown to be associated with vessel wall remodeling (11–14) and elastic fiber thinning (15) in BAV patients. Thus, 4D flow can provide additional information on cardiovascular pathologies, which influence cardiac hemodynamics, and may help to guide therapy. The aim of this study was to investigate aortic hemodynamic changes over time using 4D flow MRI in patients with aortic valve stenosis and/or bicuspid aortic valve with and without aortic valve replacement.

## 2. Materials and methods

### 2.1. Study population

**Figure 1:**
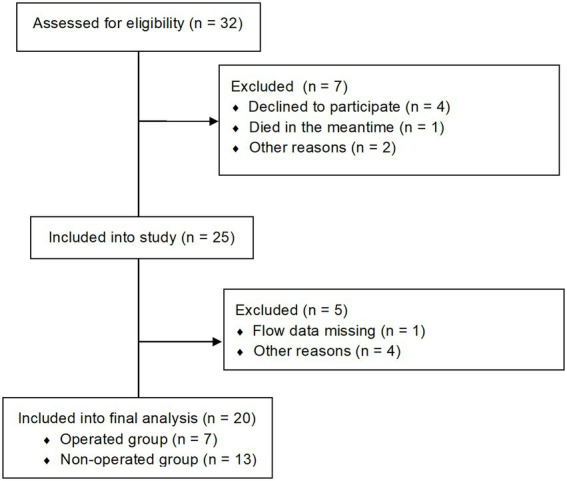
Consort flow diagram of patients enrollment.

Participants were initially prospectively recruited (2, 16) and then asked to come back for a follow up investigation. A total of 20 patients (14 with BAV and 6 with TAV and AS) could be included for follow up aortic 4D flow MRI examinations, resulting in a total of 40 MRI datasets. The diagnosis of AS was based on the aortic orifice area (AOA). Most patients (14/20) reported at least one symptom (palpitations, dizziness, syncope or dyspnea according to the NYHA classification) of AS at follow up (for further information see Supplementary Table S1). A total of 7 patients (1 patient with TAV and 6 patients with BAV) received an aortic valve replacement between baseline (B) and follow up (FU) (operated group = OP), 13 patients did not (= non-operated group = NOP). The enrollment is shown in Figure 1. A total of 3 BAV patients had a right-coronary/non-coronary (RN) cusp fusion (one in the OP group, two in the NOP group), 11 a right-coronary/left-coronary (RL) cusp fusion. For each participant, written informed consent was obtained before the study, after approval by the local ethical committee (Charité ethical approval EA 1/135/17 on August 10, 2017). The study was registered at ISRCTN (ISRCTN17935517).

### 2.2. Cardiovascular magnetic resonance imaging acquisition

All cardiovascular MRI examinations at both time points were performed on a 3 Tesla Scanner (MAGNETOM Verio; Siemens Healthineers GmbH, Erlangen, Germany). 4D flow cardiovascular MRI data were acquired using a sagittal oblique volume covering the thoracic aorta. Prospective ECG gating was used in combination with a respiratory navigator placed on the lung–liver interface to allow data acquisition during free breathing. The following scan parameters were used: echo time = 2.6 ms, repetition time = 5.1 ms, bandwidth = 450 Hz/pixel, imaging acceleration using PEAK GRAPPA with a reduction factor of R = 5, net acceleration 4.17, reference lines = 20, flip angle α = 7° to 9°, temporal resolution = 40.8 ms, field of view = 360 mm × 270 mm, voxel size = 2.7 mm × 2.3 mm × 2.6 mm, phase encoding direction = anterior–posterior, number of slices = 32, encoding velocity = 1.5–2.5 m/s.

Additionally, standard steady-state free-precession cine images were acquired for cardiac chamber quantification and for planimetry of the AOA. Imaging parameters were as follows: repetition time = 3.1 ms, echo time = 1.3 ms, flip angle = 45°, field of view = 276 mm × 340 mm, matrix = 156 × 192, slice thickness = 6 mm (chambers) and 5 mm (aortic valve), bandwidth = 704 Hz/px, parallel imaging using GRAPPA reconstruction with R = 2, 30 cardiac phases. Cardiac chamber and AOA quantification was performed using CVI42 (Circle Cardiovascular Imaging, Calgary, Canada). Maximum aortic area was defined as the product of the two orthogonal aortic diameter measurements.

### 2.3. Four dimensional flow image analysis

First, 4D flow MRI data were corrected for Maxwell terms and eddy-currents (MATLAB, The MathWorks Inc., United States) (17). Aliasing correction was applied in all cases as part of a standardized approach. If correction failed, the respective plane was excluded from analysis. Manual segmentation of the aorta was performed (Mimics, Materialise, Belgium). Helical and vortical blood flow patterns in the ascending aorta were semi-quantitatively evaluated using pathline movies and graded as follows: 0 (none), 1 (flow rotations<360°), 2 (>360°) and 3 (>360° with increased flow) (18). A vortical flow formation was defined as revolving particles around a point within the vessel with a rotation direction deviating by >90° from the main physiological flow direction. A helical flow formation was considered as a regional fluid circulation around an axis parallel to bulk fluid motion (i.e., along the longitudinal axis of the vessel), thereby creating a corkscrew-like motion) (2).

**Figure 2:**
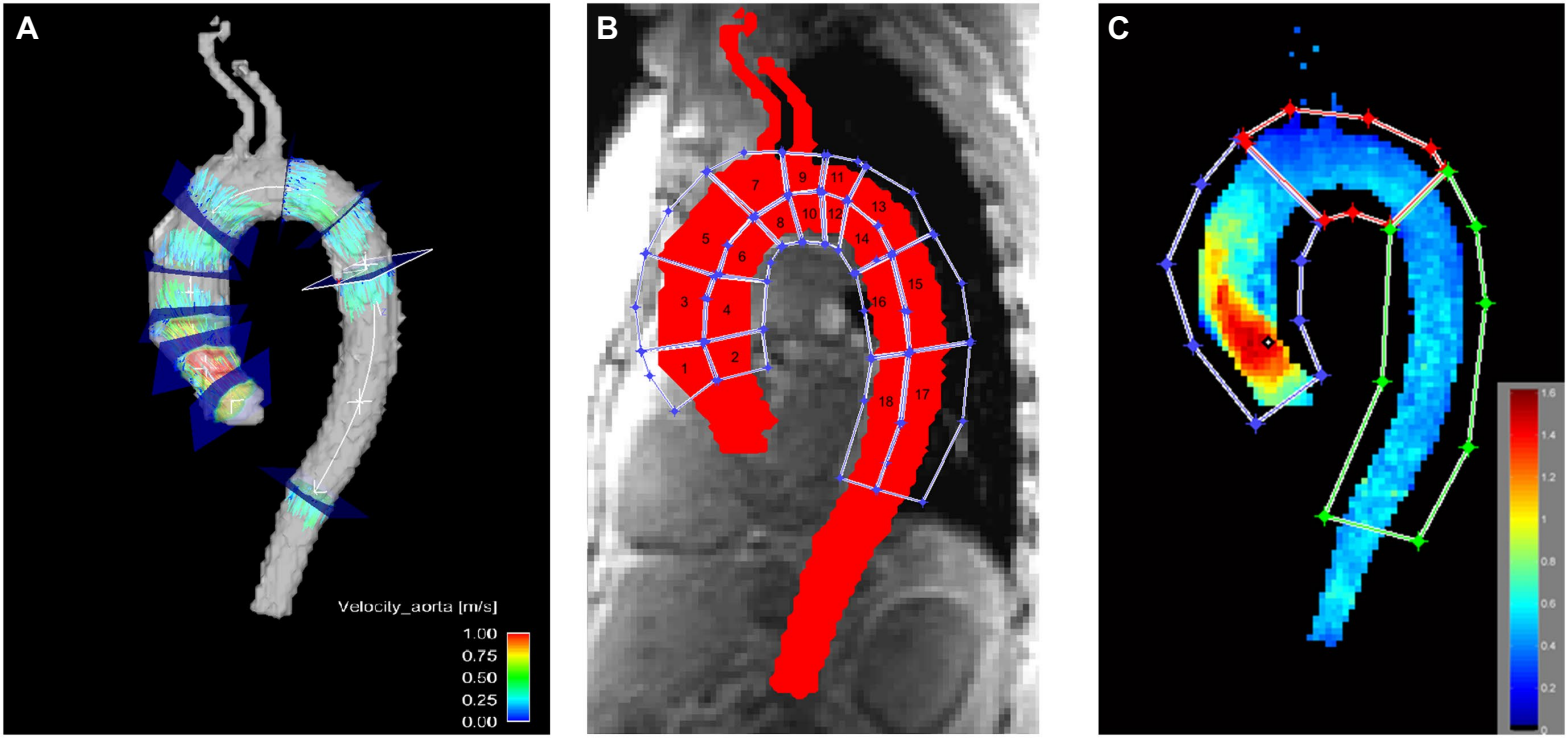
Visualization of the locations used for quantitative assessment in the thoracic aorta. **(A)** Flow was evaluated in nine axial planes in the aorta. **(B)** WSS was evaluated in 18 aortic wall regions. **(C)** Peak velocity was evaluated in the ascending aorta, the aortic arch and the descending aorta. Notice the elevated peak velocity in the ascending aorta due to the pathologic aortic valve.

Then, 3D blood flow visualization and positioning of 2D cross-sectional planes for flow quantification were conducted (EnSight, Version 10.0, CEI, Apex, NC, United States). Nine planes were positioned perpendicular to the longitudinal axis of the aortic wall at the following locations: in the left ventricular outflow tract (P1), at the level of the sinus of valsalva (P2), at the sinotubular junction (P3), in the mid-ascending aorta (P4), defined as the level at which the pulmonary trunc divided into the left and right pulmonary artery, proximal to the brachiocephalic trunk (P5), in the mid-aortic arch between the left common carotid artery and the left subclavian artery (P6), distal to the left subclavian artery (P7), in the proximal descending aorta at the level of P4 (P8), and in the distal descending aorta (P9; Figure 2A).

3D WSS was calculated for the entire thoracic aortic wall at peak systole using a home built analysis tool (MATLAB, The MathWorks Inc., United States), as described previously by Potter et al. (19) and van Ooij et al. (20). Briefly, based on the 3D segmentation of the thoracic aorta, systolic 3D WSS along the entire aortic wall was calculated from 4D flow velocity data. The WSS vector was estimated at the wall based on the 3D spatial velocity gradient perpendicular to the vessel wall. Systolic 3D WSS vectors were then calculated by averaging WSS vectors for five timeframes centered on peak systole (defined as the cardiac timeframe with the highest average velocity in the aorta segmentation). Finally, the absolute WSS (length of WSS vector) was calculated. For WSS calculation the whole area of the Thoracic aorta was covered by regions in a three-dimensional aspect and chosen according to surgical aspects relevant for aortic valve replacement or aortic replacement (6). WSS was calculated in the resulting 18 regions (Figure 2B). Additionally, the aorta was divided into ascending aorta, aortic arch and descending aorta as well as in the inner part and the outer part for WSS evaluation.

Peak velocities were obtained from velocity maximum intensity projections in the ascending aorta, the aortic arch and the descending aorta (MATLAB, The MathWorks Inc., United States; Figure 2C) (21). In short, an aortic velocity field was used to generate a velocity maximum intensity plot spanning three time frames during peak systole in sagittal, coronal and axial views.

### 2.4. Statistical analysis

Statistical analysis was performed using SPSS version 25 (IBM, Armonk, NY, United States). Parameters were compared between B and FU for all patients as well as between the OP group and the NOP group. Parameters at B and FU were compared using Wilcoxon Signed Rank Test, parameters between groups were compared using Mann–Whitney U Test as the tested cohort was too small to reach normal distribution. Comparisons between parameters in the ascending aorta, the aortic arch and the descending aorta were performed using Friedman Test, for pairwise comparisons Wilcoxon Test was used. Nominally scaled data were compared using Chi Square Test.

## 3. Results

Mean duration from B to FU was 4.3 ± 1.4 years in the OP group and 4.4 ± 1.5 years in the NOP group. Operated patients were significantly older than non-operated patients (age at FU: OP group 73.3 ± 4.4 years, NOP group 57.5 ± 15.9 years, *p* < 0.05) and had a significantly lower Body Mass Index (BMI) (BMI at FU: OP group 25.0 ± 2.1 kg/m^2^, NOP group 27.8 ± 3.2 kg/m^2^, *p* < 0.05). Left ventricular (LV) ejection fraction (EF) and LV mass significantly decreased in operated patients from B to FU (B LV-EF: 69 ± 5.7%, FU LV-EF: 63.7 ± 4.9%, p < 0.05; B LV mass: 203.3 ± 90.1 g, FU LV mass: 153.4 ± 46.7 g, p < 0.05), even after adjusting the latter for body surface area (BSA) (B LV mass/BSA: 105.9 ± 41.3 g/m^2^, FU LV mass/BSA: 81.6 ± 20.1 g/m^2^, p < 0.05; for patient characteristics see Table 1). Maximal aortic area, the product of the two orthogonal aortic diameter measurements, did neither differ between NOP and OP group nor between time points (Table 1). Flow volume measurements within P1 planes could not reliably be evaluated due to positioning of the field of view or artifacts and were therefore excluded from further analysis. In patients with a replaced aortic valve at FU, results between the valve and 2 cm above were not included in the analysis as those numbers were not reliable due to local magnetic field distortions. This applied to the sinotubular junction (22). A total of 72 planes analyzed across all patients at baseline and follow-up, aliasing correction did not work. Therefore, these planes were not included in the final analysis.

**Table 1:** Patients’ characteristics at baseline (B) and follow up (FU).

	Non-operated patients at B	Non-operated patients at FU	Operated patients at B	Operated patients at FU
Sex (female/male)		5/8		1/6
Age (years)		57.5 ± 15.9		73.3 ± 4.4
Height (cm)	172.5 ± 10.7	172.6 ± 9.5	170.6 ± 7.1	170.7 ± 7.8
Weight (kg)	82.6 ± 13	83.2 ± 13.7	75.1 ± 9.8	72.9 ± 8.5
BMI (kg/m^2^)	27.8 ± 4^+^	27.8 ± 3.2^+^	25.8 ± 2.7^+^	25.0 ± 2.1^+^
Systolic blood pressure (mmHg)	135.3 ± 10.9	125.7 ± 17.5	147.2 ± 8.2	136 ± 17.9
Diastolic blood pressure (mmHg)	80.6 ± 16.3	75.7 ± 13.5	88.2 ± 8.1	72.3 ± 7.1
Heart rate (bpm)	68.2 ± 11.7	68.7 ± 13	66.7 ± 8.2	64 ± 10.1
Max. aortic area (cm^2^)	1.25 ± 0.43	1.28 ± 0.442	1.15 ± 0.25	1.04 ± 0.24
Max. aortic diameter (mm)	39.3 ± 6.7	39.8 ± 7.0	38.1 ± 4.3	36.2 ± 4.3
Aortic valve area (cm^2^)	2.0 ± 0.5	2.0 ± 0.7	0.8 ± 0.2	1.9 ± 0.2
Aortic valve area index (cm^2^/m^2^)	1.0 ± 0.3	1.0 ± 0.3	0.4 ± 0.1	1.0 ± 0.1
LV-EDV (mL)	153 ± 34.2	151.5 ± 31.6	133.1 ± 13.6	131.113.5
LV-EDV/BSA (mL/m^2^)	76.7 ± 14.5	76.2 ± 15.5	70.7 ± 5.6	70.6 ± 5.5
LV-SV (mL)	91.9 ± 17	92.5 ± 18.1	91.9 ± 6.2	83.3 ± 6.2
LV-SV/BSA (mL/m^2^)	46.3 ± 7.3	46.6 ± 8.8	48.9 ± 4	45 ± 4.5
LV-EF (%)	60.9 ± 5.7	61.7 ± 5.6	69 ± 5.7*	63.7 ± 4.9*
LV-mass (g)	151.5 ± 41.2	148.8 ± 39.5	203.3 ± 90.1*	153.4 ± 46.7*
LV-mass/BSA (g/m^2^)	75.6 ± 17.6	74.5 ± 18.2	105.9 ± 41.3*	81.6 ± 20.1*

+ indicates a significant differen*c*e (*p* < 0.05) between operated and non-operated patients, * indicates a significant difference (*p* < 0.05) between B and FU. LV, left ventricular; EDV, enddiastolic volume; BSA, body-surface-area; SV, stroke volume; EF, ejection fraction.

### 3.1. Overall findings

**Figure 3:**
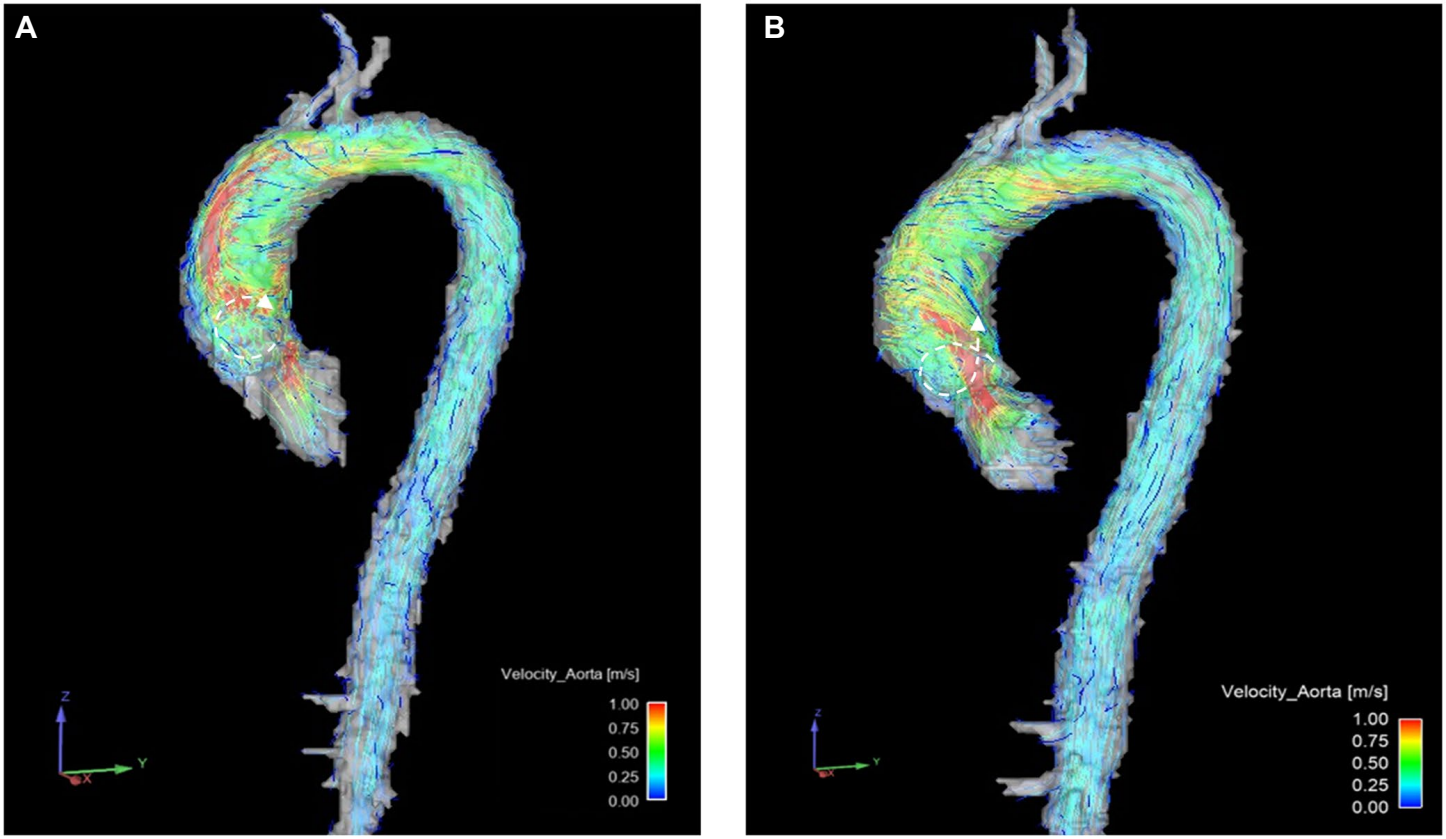
Example of helical (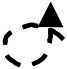) and vortical (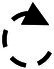) flow formations changing in one patient before **(A)** and after **(B)** surgery. Notice the reduction of vortical flow formations between **(A,B)** and the increase of helical flow formations.

Most patients showed vortical and/or helical flow formations. There was no significant change regarding vortical and helical flow formations between B and FU (as an example see Figure 3).

**Figure 4:**
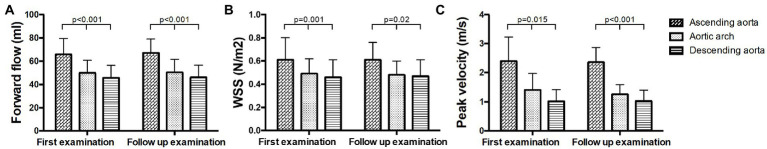
Hemodynamic measurements in the ascending aorta, the aortic arch and the descending aorta at baseline and follow up. **(A)** Forward flow volumes, **(B)** WSS, and **(C)** peak velocity.

Forward flow volume was highest in the ascending aorta and decreased in arch and descending aorta in all examinations (*p* < 0.001, Figure 4A; Supplementary Table S2A). Forward flow volumes at P5 increased significantly at FU compared to B (B 62.01 ± 11.6 ml vs. FU: 66.65 ± 11.38 ml, *p* = 0.035). WSS was significantly higher in the ascending aorta than in the aortic arch and the descending aorta in all patients at both examinations (*p* < 0.05, Figure 4B; Supplementary Table S2B). Peak velocity decreased over the course of the aorta from the ascending aorta to the descending aorta at both time points as well (p < 0.05, Figure 4C; Supplementary Table S2C).

### 3.2. Hemodynamic changes in non-operated patients

**Figure 5:**
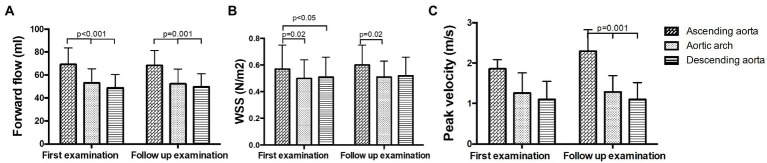
Hemodynamic measurements in non-operated patients in the ascending aorta, the aortic arch and the descending aorta at baseline and follow up. **(A)** Forward flow volumes, **(B)** WSS, and **(C)** peak velocity.

No significant changes in forward flow volumes were found between B and FU. Forward flow volume was significantly higher in the ascending aorta than in the aortic arch and the descending aorta at both time points (*p* < 0.05, Figure 5A; Supplementary Table S3A). No significant changes in WSS were observed between B and FU. WSS did not decrease significantly over the whole aorta. However, in pairwise comparisons, significant differences between the ascending aorta and the aortic arch (B and FU) or descending aorta (only at B) could be found (*p* < 0.05, Figure 5B; Supplementary Table S3B). No significant changes in peak velocity were found between B and FU. Peak velocity significantly decreased throughout the aorta which led to significant differences only at FU (*p* = 0.001, Figure 5C; Supplementary Table S3C).

### 3.3. Hemodynamic changes in operated patients

**Figure 6:**
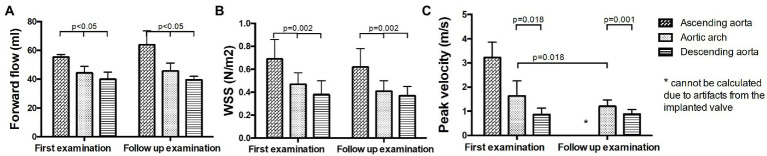
Hemodynamic measurements in operated patients in the ascending aorta, the aortic arch and the descending aorta at baseline and follow up. **(A)** Forward flow volumes, **(B)** WSS, and **(C)** peak velocity.

No significant changes in forward flow volumes were found between B and FU. Forward flow volume was significantly higher in the ascending aorta than in the aortic arch and the descending aorta at both time points (*p* < 0.05, Figure 6A; Supplementary Table S4A). No significant changes in WSS were found between B and FU. WSS was significantly higher in the ascending aorta than in the aortic arch and the descending aorta at both examinations (*p* < 0.05, Figure 6B; Supplementary Table S4B). No statement for changes in peak velocity in the ascending aorta at FU can be made, as due to the artifacts from the implanted valve, it would not be reliable. Peak velocity decreased at FU compared to B in the aortic arch (*p* = 0.018, Figure 6C; Supplementary Table S4C). Peak velocity was higher in the aortic arch than in the descending aorta at both examinations (*p* = 0.018, Figure 6C; Supplementary Table S4C).

### 3.4. Comparison between non-operated patients and operated patients

Forward flow volumes were significantly lower in the ascending aorta in the OP group than in the NOP group at B (NOP 69.32 mL ± 14.19 mL vs. OP 55.38 mL ± 1.85 mL *p* = 0.029), while no significant difference could be found anymore at FU. WSS in the outer ascending aorta was significantly higher in the OP group than in the NOP group at B (NOP 0.57 ± 0.18 N/m^2^ vs. OP 0.69 ± 0.17 N/m^2^, *p* = 0.008) as well as in segments 3 and 5 (segment 3: OP 0.94 ± 0.25N/m^2^, NOP 0.71 ± 0.25N/m^2^, *p* = 0.018; segment 5: OP 0.73 ± 0.2N/m^2^, NOP 0.52 ± 0.13 N/m^2^, *p* = 0.024), while at FU no significant difference could be found. WSS in the descending aorta was higher in the NOP group than in the OP group at B and at FU (NOP B: 0.51 ± 0.15 N/m^2^ FU: 0.52 ± 0.14 N/m^2^; OP B: 0.38 ± 0.12 N/m^2^ FU: 0.37 ± 0.08 N/m^2^, both *p* < 0.05).

## 4. Discussion

This study investigated changes in hemodynamics of patients with aortic valve pathologies over a mean duration of 4 years using 4D flow MRI. One group of the patients had an aortic valve replacement during the course of the study, the other group did not.

Our main findings were: (i) patients who received an aortic valve replacement between baseline and follow up (OP group), had a higher WSS along the outer curvature of the ascending aorta prior to the surgery than patients who did not undergo surgery (NOP group), (ii) peak velocity decreased after aortic valve replacement and (iii) patients, who received an aortic valve replacement between baseline and follow up, had initially a lower forward flow in the ascending aorta than patients, who did not receive an aortic valve replacement.

Patients, who had an aortic valve replacement during the course of the study, had a higher WSS in the outer ascending aorta than non-operated patients, but only at baseline before surgery. Afterwards, there was no difference in WSS anymore, as WSS in the OP group decreased and WSS in the NOP group slightly increased. A higher WSS in patients with aortic stenosis than in healthy volunteers was previously described by van Ooij et al. (20). They found an increased WSS in the outer ascending aorta in 166 patients with AS and 210 patients with a BAV (3), which is the same location that we could identify in our study. Elevated WSS of patients with BAV was also reported by Farag et al. (23), who further showed a negative correlation between aortic diameter and increased WSS. Rahman et al. (6) investigated BAV patients over 3 years and found a decrease in WSS with a minimal increase in aortic dilatation as well. In our study, we did not find a correlation between WSS and aortic diameter, as with decrease of WSS the aortic diameter did not significantly change. However, we only observed a decrease in WSS in patients who underwent surgery during the course of the study. The procedure might have influenced hemodynamics enough in order to reduce WSS without a compulsory growth of the aorta. Additionally, we also included TAV patients with AS, who might have a different pathologic pathway leading to aortic remodeling. BAV patients have a faster aortic growth than TAV patients before aortic valve replacement, but not after (24). A decrease of WSS in patients with aortopathy undergoing aortic valve replacement was also found by Bollache et al. (8), which is in line with our findings.

Peak velocity decreased in the OP group at follow up compared to baseline examination in the aortic arch; values in the OP group adapt almost to the NOP group. In the follow up of BAV patients over 3 years by Rahman et al. (6) an increased peak velocity over time was found. This increase was associated with worsening of the valve function. We, however, found a decrease of peak velocity in patients during follow up, but only in those who had aortic valve replacement. These findings are in line, as valve replacement relieves valvular dysfunction.

Although there was no significant increase in forward flow volumes before and after surgery, there was a significantly lower flow in the ascending aorta at baseline in the OP group than in the NOP group, which could not be seen at follow up due to an (non-significant) increase in flow volumes in the operated group. Kamada et al. (25) found a significant increase in flow volumes in the ascending aorta after aortic valve replacement and a decrease of the flow angle, which supports our findings. We did, however, not find a significant change in helical and vortical flow pattern in our patient cohort, but only changes in single patients. The number of patients in our study might be too small to reach statistical significance in this regard. Moreover, forward flow volume was highest in the ascending aorta and decreased in arch and descending aorta in all examinations. We did not investigate the reason for this observation. One reason might be that this is due to the supra-aortic vessels bringing blood to the head. Future studies with new 4D flow sequences with a higher spatial resolution to analyze blood flow parameters in the Carotid arteries are needed to test this hypothesis.

It has been shown that aortic hemodynamics and the diameter of the ascending aorta in BAV patients depend on the morphology of the valve, i.e., on the type of cusp fusion (26, 27). We did not differentiate between the different bicuspid aortic valve morphologies in our study as the majority of patients in both groups (OP and NOP) had a RL cusp fusion and no difference in maximum aortic area was found. Additionally, in patients with aortic stenosis, differences in alterations of WSS between different valve morphologies are no longer apparent (3).

In the descending aorta we found significantly higher WSS in the NOP group than in the OP group. This might be due to the fact that the patients in the NOP group were significantly younger than patients in the OP group. WSS dependency on age with lower WSS at higher age has been shown in normal volunteers (28) and patients with bicuspid aortic valves (29). This might explain our findings of a higher WSS in the descending aorta in the NOP group.

### 4.1. Limitations

This study comprises a small number of patients in total and with aortic valve replacement between baseline and follow-up studies, which was due to the long follow up period and the age of the included patients. Additionally, the cohort was heterogenous in its clinical conditions, which might be the reason for the absence of differences between baseline and follow-up. It was conducted at one site only. Multicenter studies are necessary to confirm these findings in a larger cohort. There are several possible sources of error in our 4D flow image analysis: Aortic helical flow was only assessed using a semi-quantitative approach.

### 4.2. Conclusion

In this 4-year follow up of patients with aortic valve pathology, cardiovascular 4D flow MRI revealed hemodynamic changes in the aorta in patients undergoing aortic valve replacement. WSS decreased and aortic forward flow volumes increased compared to patients who were not operated. Aortic valve replacement improves aortic hemodynamics and might decelerate aortic wall remodeling.

## Data availability statement

The datasets presented in this article are not readily publically available due to German Data Protection Laws. Requests to access the datasets should be directed to the corresponding author.

## Ethics statement

The studies involving human participants were reviewed and approved by Charité Ethics Committee, Charité – Universitätsmedizin Berlin, Berlin, Germany. The patients/participants provided their written informed consent to participate in this study.

## Author contributions

SW: conceptualization, formal analysis, investigation, methodology, project administration, validation, visualization, and writing – original draft. AM: data curation and investigation. RH: investigation, formal analysis, and writing – review and editing. TM: data curation. RT and FK-B: investigation, resources, and writing – review and editing. EB and MM: resources, software, and writing – review and editing. JS-M: conceptualization, methodology, project administration, resources, supervision, and writing – review and editing. All authors read and approved the final manuscript.

## Conflict of interest

The authors received research support from Siemens Healthineers (JS-M, SW, and MM) and Circle Cardiovascular Imaging (JS-M and SW), research grants from Circle Cardiovascular Imaging (MM) and Cryolife Inc (MM), and performed activities in consulting Circle Cardiovascular Imaging (MM).

The remaining authors declare that the research was conducted in the absence of any commercial or financial relationships that could be construed as a potential conflict of interest.

## Publisher’s note

All claims expressed in this article are solely those of the authors and do not necessarily represent those of their affiliated organizations, or those of the publisher, the editors and the reviewers. Any product that may be evaluated in this article, or claim that may be made by its manufacturer, is not guaranteed or endorsed by the publisher.
