# Supplementary material for: Changes of aortic hemodynamics after aortic valve replacement—A four dimensional flow cardiovascular magnetic resonance follow up study
Source: Front Cardiovasc Med. 2023 Feb 14;10:1071643. doi: 10.3389/fcvm.2023.1071643 (PMC9971963; doi:10.3389/fcvm.2023.1071643)
Supplement: Supplementary file 1 [file Data_Sheet_1.PDF]

Supplementary Material

1.1 Supplementary Tables

Supplementary Table 1

Symptoms of aortic stenosis at follow up in patients without replaced aortic valve during the course of the study (NOP-group) and patients with aortic valve replacement (OP-group).

	Palpitations	Dizziness	Syncope	Dyspnea
OP-group	2	1	0	5
NOP-group	2	4	2	7
Total	4	5	2	12

Supplementary Table 2

Hemodynamic measurements in the ascending aorta, the aortic arch and the descending aorta at baseline and follow up for a) forward flow volumes, b) WSS and c) peak velocity.

a)

	Ascending aorta (ml)	Aortic arch (ml)	Descending aorta (ml)
First examination	65.83 ± 13.68*	49.97 ± 10.85*	45.83 ± 10.47*
Follow-up examination	67.11 ± 11.99†	50.37 ± 11.19†	46.14 ± 10.41†

* = p<0.001, † = p<0.001

b)

	Ascending aorta (N/m^2)	Aortic arch (N/m^2)	Descending aorta (N/m^2)
First examination	0.61 ± 0.19*	0.49 ± 0.13*	0.46 ± 0.15*

Follow-up examination	0.61 ± 0.15†	0.48 ± 0.12†	0.47 ± 0.14†

* = p=0.001, † = p=0.02

c)

	Ascending aorta (m/s)	Aortic arch (m/s)	Descending aorta (m/s)
First examination	2.4 ± 0.83*	1.41 ± 0.56*	1.02 ± 0.4*
Follow-up examination	2.36 ± 0.51†	1.26 ± 0.33†	1.03 ± 0.37†

* = p=0.015, † = p<0.001

Supplementary Table 3

Hemodynamic measurements in non-operated patients the ascending aorta, the aortic arch and the descending aorta at baseline and follow up for a) forward flow volumes, b) WSS and c) peak velocity.

a)

	Ascending aorta (ml)	Aortic arch (ml)	Descending aorta (ml)
First examination	69.32±14.19*	53.19±12.28*	48.91±11.52*
Follow-up examination	68.46±13†	52.38±12.84†	49.64±11.44†

* = p<0.001, † = p=0.001

b)

	Ascending aorta (N/m^2)	Aortic arch (N/m^2)	Descending aorta (N/m^2)
First examination	0.57±0.18*$^{+}$	0.5±01.4*	0.51±0.15^{+}
Follow-up examination	0.6±0.15†	0.51±0.12†	0.52±0.14

* = p=0.02, $^{+}$ = p<0.05, † = p=0.02

c)	Ascending aorta (m/s)	Aortic arch (m/s)	Descending aorta (m/s)
First examination	1.86±0.23	1.26±0.5	1.1±0.45
Follow-up examination	2.3±0.53*	1.29±0.4*	1.1±0.42*

* = p=0.001

Supplementary Table 4

Hemodynamic measurements in operated patients the ascending aorta, the aortic arch and the descending aorta at baseline and follow up for a) forward flow volumes, b) WSS and c) peak velocity.

a)	Ascending aorta (ml)	Aortic arch (ml)	Descending aorta (ml)
First examination	55.38±1.85*	44.44±4.48*	40.11±4.83*
Follow-up examination	63.88±9.6†	45.79±5.4†	39.63±2.42†

* = p<0.05, † = p<0.05

b)	Ascending aorta (N/m^2)	Aortic arch (N/m^2)	Descending aorta (N/m^2)
First examination	0.69±0.17*	0.47±0.10*	0.38±0.12*
Follow-up examination	0.62±0.16†	0.41±0.09†	0.37±0.08†

* = p=0.002, † = p=0.002

c)

	Ascending aorta (m/s)	Aortic arch (m/s)	Descending aorta (m/s)
First examination	3.22±0.64	1.64±0.61*#	0.87±0.26*
Follow-up examination	+	1.2±0.27†#	0.88±0.19†

+ cannot be calculated due to artifacts from the implanted valve * = p=0.018,

† = p =0.001, # = p =0.018
