# Supplementary material for: Changes of aortic hemodynamics after aortic valve replacement—A four dimensional flow cardiovascular magnetic resonance follow up study
Source: Front Cardiovasc Med. 2023 Feb 14;10:1071643. doi: 10.3389/fcvm.2023.1071643 (PMC9971963; doi:10.3389/fcvm.2023.1071643)
Supplement: Supplementary file 2 [file Data_Sheet_2.PDF]

Supplementary Material

Description of the WSS methodology

The following is a description of the method applied to determine the wall shear stress (WSS), taken from a previously published article (1).

Data analysis

Pre-processing and 3D segmentation – All 4 D flow MRI data were corrected for eddy currents, Maxwell terms and velocity aliasing using in-house built software in Matlab (Natick, The Mathworks, USA) (25). 3D Phase contrast (PC) magnetic resonance angiography (MRA) images were created by voxel-wise multiplication of the magnitude data with absolute velocities averaged over all cardiac time frames (25). The thoracic aorta was semi-automatically segmented (Mimics, Materialise, Leuven, Belgium). Peak systole was defined as the time frame with the highest absolute veloxity averaged over the segmentation.

WSS estimation – For WSS calculation, all segmentations were smoothed using a Laplacian filter (26) to obtain a smooth surface of the aortic wall. 3D WSS along the aortic lumen surface was calculated as described previously by Potters et al. (18). Briefly, for each time frame within the cardiac cycle WSS estimation was based on the shear stress tensor

$$\vec{\tau} = 2 \cdot \eta \cdot \dot{\varepsilon} \cdot \vec{n}$$

With η the dynamic viscosity (Newtonian 3.2 x·10^{-3} Pa·s), ε the rate of deformation tensor and $\vec{n}$ the normal vector orthogonal to the vessel wall. This equation can be simplified by rotating the aorta such that the z-axis (0,0,1) is aligned with the normal vector of the aortic wall: $\vec{n} = (0,0,1)$. Assuming that no flow occurred through the aortic wall, $\vec{n} \cdot \vec{v} = 0$ at the wall), the inner product of the rate of deformation tensor and the normal vector is reduced to:

$$2 \cdot \dot{\varepsilon} \cdot \vec{n} = \left(\frac{\partial vx'}{\partial z'}, \frac{\partial vy'}{\partial z'}, 0\right)$$

with $\frac{\partial vx'}{\partial z'}$ and $\frac{\partial vy'}{\partial z'}$ the shear rates or spatial velocity derivatives at the wall in the rotated coordinate system. The rotated WSS vector $\vec{\tau}'$ was then defined as:

$$\vec{\tau}'_x = \eta \cdot \frac{\partial \dot{v}x'}{\partial z'}, \vec{\tau}'_y = \eta \cdot \frac{\partial \dot{v}y'}{\partial z'}, \vec{\tau}'_z = 0$$

and the shear rates were derived from 1D smoothing splines (27) fitted through the rotated x- and y-velocity values along the inward normal vector. Subsequently, the WSS vector was transformed to the original coordinate system by inverse rotation. Systolic 3D WSS vectors were then calculated by averaging WSS vectors for five time frames centred on peak systole (defined as the cardiac time frame with the highest average velocity in the aorta segmentation).

References

1. van Ooij P, Powell AL, Potters WV, Carr JC, Markl M, Barker AJ. Reproducibility and interobserver variability of systolic blood flow velocity and 3D wall shear stress derived from 4D flow MRI in the healthy aorta. J Magn Reson Imaging. 2016;43(1):236-48.
